# Supplementary material for: The relationship between modifiable lifestyle behaviours and self-reported health in children and adolescents in the United Kingdom
Source: PLoS One. 2024 May 16;19(5):e0303575. doi: 10.1371/journal.pone.0303575 (PMC11098376; doi:10.1371/journal.pone.0303575)
Supplement: Attachment — Submitted filename: Response to reviewers.docx [file pone.0303575.s001.docx]

**Reviewer #1**: Thank you for the opportunity to review this paper who analyzed the relationship between specific easily modifiable lifestyle factors and self-reported health in Children and Adolescents in the United Kingdom. The date is from Millennium Cohort Study, specifically the fifth sweep in 2015. The study's utilization of multiple regression analysis provides a robust framework for examining these associations, offering valuable insights into potential avenues for promoting healthier behaviors and preventing chronic diseases.

**Response**: We would like to sincerely thank the reviewer for the time they have taken to review our manuscript. We greatly appreciate the recommendations that have been made and we have responded in an itemised fashion in this document, highlighting the changes in the manuscript.

**Reviewer #1:**The authors should move table 1 in the section Results, now is placed before the Statistical Analysis.

**Response**: We have now added this to results section.

**Reviewer #1:** Overall, this paper makes a significant contribution to the literature on lifestyle factors and health outcomes in young individuals. However, it would be beneficial for the authors to provide additional insights into the limitations of the study, such as potential confounding variables or biases, which could impact the generalizability of the findings.

**Response**: We do have an already extensive limitations section but have now added:

*“Lastly, multiple regression analysis may not sufficiently capture the complexities and potentially non-linear interactions of health behaviours in adolescents. Such behaviours are multifaceted and variable, particularly in a dynamically developing population. The use of linear models could oversimplify these behaviours; thus, while our study provides valuable insights, interpretations of the relationships between lifestyle factors and self-reported health are constrained by certain methodological limitations.”*

**Reviewer #1:** Additionally, suggestions for future research directions would further enhance the paper's contribution to the field.

**Response**: We have now added:

*“Accordingly, future research should focus on establishing how long-term compliance can be encouraged, perhaps by incorporating technology and gamification into the execution of healthy life behaviours in children and adolescents thus making such activities more fun and engaging.”*

**Reviewer #1:** The references should be check by the authors as some of them are not in the Journal format and they are written in different styles.

**Response**: We have now standardised and corrected the references.

**Reviewer #2**: The manuscript provides the findings of a study conducted with the objective to determine the interdependence of modifiable lifestyle factors on self-reported health in British adolescents. The theoretical basis is well founded and justifies the study. The bibliographic references are current and relevant to the subject of study. The methods used are sufficiently described, appropriate and consistent with the study proposal. The proposal is presented in an attractive manner, and the discussion of the findings show good prospects to the knowledge area. The conclusions are consistent with presented evidence and arguments. The findings of the study have good prospects to offer important contributions to the knowledge area, and offers important support for the design and conduct of future studies.

**Response**: We are very grateful to the reviewer for dedicating the time to critiquing our manuscript. The insightful suggestions have been valuable and we have carefully addressed each one, noting the corresponding modifications within the manuscript.

**Reviewer #2**: However, with the intention of helping to improve the manuscript, I suggest adjusting two items:

Table 1 - Descriptive Statistics for Participant Characteristics: data related to self-reported health and modifiable lifestyle behaviors should not be presented through mean and standard deviation, but rather through prevalence rates (%) and respective confidence intervals (CI 95%) for each categorical answers.

**Response**: We have now replaced Table 1 with the frequency table from our statistical analysis, altered for publication standards.

**Reviewer #2**: The use of the term "Easily-Modifiable Lifestyle Habits": I understand and studies have shown that it is eventually possible to modify lifestyle habits, particularly in childhood and adolescence. However, this is an "extremely difficult" task, and not as the authors mention, "easily". Furthermore, there are important conceptual differences between "Habits" and "Conducts". Therefore, specifically in this study, I believe that the use of the term "Easily-Modifiable Lifestyle Habits" at various times in the text is not the most appropriate. I suggest replacing the term with "Modifiable Lifestyle Behaviors".

**Response**:

We agree fully with the reviewer and we have adjusted the language used throughout the text. We now use: ‘modifiable lifestyle behaviours’ as per the reviewer’s recommendation.
